# Effects of Dual-Task Training on Gait Ability in Older Adults with Mild Cognitive Impairment: A Randomized Controlled Trial Focused on Obstacle Negotiation

**DOI:** 10.3390/s26113415

**Published:** 2026-05-28

**Authors:** Su-Ha Lee, Chang Ho Song

**Affiliations:** Department of Physical Therapy, Sahmyook University, 26-19, Gongneung-dong, Nowon-gu, Seoul 139240, Republic of Korea; dmddo1009@naver.com

**Keywords:** mild cognitive impairment, sensor-based gait analysis, optical gait assessment, dual-task gait training, obstacle negotiation, adaptive gait, executive function, rehabilitation, CRIS, KCT0011516

## Abstract

**Highlights:**

**What are the main findings?**

- Optical sensor-based gait assessment showed that dual-task gait training with visual adaptation improved spatiotemporal gait performance in older adults with MCI.
- The largest gains occurred during level walking and predictable obstacle negotiation, whereas unpredictable obstacle conditions showed more selective improvements in adaptive gait control.

**What are the implications of the main findings?**

- Technology-assisted dual-task rehabilitation may improve balance, executive function, and fall-related confidence in older adults with MCI.
- Sensor-derived gait metrics may help quantify treatment effects during complex walking tasks and support digitally enabled gait rehabilitation research.

**Abstract:**

Older adults with mild cognitive impairment (MCI) often show gait impairment during dual-task walking and obstacle negotiation. This assessor-blinded randomized controlled trial investigated whether dual-task gait training with visual adaptation, added to a general exercise program, improves gait and related functional outcomes in older adults with MCI. Forty participants aged 65 years or older who met the MCI criteria were randomly allocated to a dual-task gait training with visual adaptation group or a control group (*n* = 20 each). Spatiotemporal and adaptive gait parameters were assessed before and after 4 weeks of intervention during level walking and during predictable and unpredictable obstacle negotiation under light and noise conditions. Balance, executive function, and concern about falling were also evaluated. Compared with the control group, the intervention group showed greater improvements in level walking and predictable obstacle negotiation, including longer step and stride length, shorter step and stride time, higher cadence, and faster gait speed. Under unpredictable obstacle conditions, gains were more selective and were observed mainly in step and stride length and adaptive gait indices. The intervention group also showed greater improvement in balance and executive function and a larger reduction in concern about falling. These findings suggest that adding dual-task gait training with visual adaptation to a general exercise program may have clinical value for improving adaptive gait and related functional outcomes in older adults with MCI. However, because the intervention group received additional gait-specific training and a higher total training dose than the control group, future dose-matched studies are needed to clarify the specific contribution of visual adaptation.

## 1. Introduction

Mild cognitive impairment (MCI) is generally regarded as an intermediate stage between normal aging and dementia, characterized by objectively detectable cognitive decline while basic independence in activities of daily living is relatively preserved [1,2]. MCI is considered a clinically meaningful stage because it carries an elevated risk of progression to dementia, and the importance of early identification and intervention has therefore been consistently emphasized [2]. More recently, MCI has been increasingly conceptualized not simply as a memory-related disorder but as a motor–cognitive condition in which cognitive decline, gait dysfunction, and postural balance impairment may interact. Compared with cognitively intact older adults, individuals with MCI have been reported to demonstrate poorer gait and balance performance, including slower gait velocity, shorter stride length, altered stride timing, and greater postural sway [3]. Because safe walking requires both forward progression and balance control, such functional vulnerability may compromise mobility and the capacity to perform activities of daily living. Accordingly, gait speed and related spatiotemporal gait parameters, together with balance ability, have emerged as key functional markers that reflect both cognitive decline and broader functional deterioration in older adults with MCI [3,4].

Although gait may appear to be an automated motor behavior, it is in fact a complex task that depends on the integration of sensory processing, postural control, adaptation to environmental demands, and continuous motor planning [5]. Among commonly used gait indices, gait speed is particularly important in geriatric assessment because walking speed tends to decline with advancing age and is closely related to stride length, cadence, and subsequent mobility decline [5,6]. Normal gait involves more than the generation of rhythmic steps; it also requires the simultaneous maintenance of balance and both reactive and anticipatory adjustments to the external environment [5]. Accordingly, age-related gait changes should be interpreted not merely as a physiological phenomenon but as a sensitive clinical marker reflecting diverse neurological and functional abnormalities. This perspective suggests that gait impairment in older adults with MCI should not be viewed solely as a problem of motor execution, but rather understood in relation to the cognitive mechanisms that regulate gait.

Under such complex gait conditions, safe mobility depends not only on physical factors such as muscle strength and joint range of motion but also on higher-order cognitive functions that support the selective processing of environmental information and the regulation of behavior. In particular, obstacle avoidance, turning, gait speed modulation, and the concurrent performance of another task while walking require appropriate allocation of attention, response selection, inhibition of irrelevant actions, and task switching. In this context, executive function is regarded as a core cognitive domain involved in planning goal-directed behavior, suppressing inappropriate responses, and shifting and monitoring behavior in accordance with changing task demands [7]. Indeed, individuals with MCI have been reported to show relatively pronounced impairments not only in delayed recall but also in visual memory and frontal lobe-based executive functioning, supporting the view that gait problems in older adults with MCI should be understood in relation to executive dysfunction as well as motor impairment [1].

The importance of executive function becomes particularly evident in obstacle negotiation, one of the more complex gait tasks. Unlike level walking, obstacle negotiation is a cognitive–motor integration task that requires individuals to scan the surrounding environment, identify the location and characteristics of an obstacle, and adapt their gait pattern accordingly to pass safely [8,9]. In other words, obstacle negotiation requires the sequential integration of visual scanning, judgments of distance and height, motor planning, and real-time gait adjustment. Moreover, as obstacle conditions become more complex, older adults tend to exhibit longer preparation times during the approach phase, suggesting that obstacle negotiation is not merely a locomotor task but one that places substantial demands on higher-order planning and control processes [9]. Accordingly, compared with level walking, obstacle negotiation may be considered a more ecologically valid assessment task because it better reflects adaptation to environmental demands and dynamic stability.

The cognitive demands of obstacle negotiation are further amplified under dual-task conditions, in which walking must be performed concurrently with a cognitive task. Because the two tasks compete for limited cognitive resources under dual-task conditions, interference may arise between gait and cognitive performance, resulting in cognitive–motor interference [7,10]. Previous studies have shown that both gait-task difficulty and cognitive-task difficulty significantly affect gait speed and stability under dual-task conditions, and that these effects are more pronounced in older adults [10]. In particular, when the walking task itself is highly attention-demanding, as in obstacle negotiation, dual-task-related gait deterioration may become more pronounced, suggesting that gait in complex environments is especially vulnerable to the depletion of cognitive resources [9,10]. In addition, older adults with MCI have been shown to exhibit poorer gait performance than cognitively unimpaired older adults under both single-task and dual-task conditions, and dual-task gait speed in particular has been proposed as a significant behavioral marker for distinguishing MCI [2]. These findings suggest that, when assessing gait or designing interventions for older adults with MCI, dual-task gait may reflect cognitive vulnerability more sensitively than single-task gait.

Meanwhile, both obstacle negotiation and dual-task conditions require continuous processing of changing environmental information and ongoing adjustment of gait, making the efficient use of sensory information essential. Among the available sensory cues, visual information plays a particularly important role in anticipating the walking path, regulating foot placement and trajectory, and modifying strategies for successful obstacle crossing [11]. In particular, environmental information obtained through the lower visual field is critical for identifying the space immediately in front of the feet, the condition of the walking surface, and the location of obstacles, and real-time adaptation to this information is a prerequisite for safe gait performance [11]. Furthermore, when obstacle negotiation is combined with a dual-task condition, increased prefrontal activation or oxygenation has been reported, suggesting that such tasks require additional cognitive resources [8]. Therefore, the ability to adapt to visual information may be a key factor in explaining dynamic stability during complex gait tasks in older adults with MCI and may also provide a theoretical basis for designing interventions aimed at improving obstacle negotiation.

However, previous studies have primarily focused on characterizing gait performance in older adults with MCI or identifying between-group differences under relatively simple level walking tasks or fixed dual-task conditions [2,10]. Although obstacle negotiation has been recognized as a gait task with high cognitive demands [8,9], and gait impairment may become more pronounced under dual-task conditions [10], evidence supporting the translation of these findings into intervention contexts remains limited [12,13,14,15]. Although intervention evidence is not yet extensive, existing studies provide preliminary support for the therapeutic potential of dual-task and obstacle-based gait training in older adults. In older adult populations without a specific MCI diagnosis, dual-task balance training has been shown to improve dual-task performance during standing postural control, although effects on conventional balance and mobility outcomes have been less consistent [12]. Obstacle-negotiation-based exercise has also been investigated as a fall-prevention approach in community-dwelling older adults, with complex obstacle negotiation exercise associated with lower fall incidence during follow-up [13]. In older adults with MCI or broader cognitive impairment, cognitive and motor dual-task walking training has been reported to improve dual-task walking performance, and meta-analytic evidence suggests that simultaneous dual-task training can improve global cognition, executive function, working memory, and gait [14,15]. Nevertheless, these studies have generally focused on standing balance, straight-path walking, or overall gait outcomes such as gait speed, whereas relatively little is known about interventions that integrate visual stimulus–response adaptation, obstacle negotiation, and predictable or unpredictable environmental conditions within a single gait-specific training program. In particular, evidence remains insufficient regarding the effects of dual-task gait training designed to enhance adaptation to visual information on obstacle negotiation performance and related functional outcomes in older adults with MCI. Accordingly, the present study aimed to investigate the effects of this training on gait performance under different obstacle negotiation conditions, as well as on executive function, balance, and falls efficacy, in older adults with MCI.

## 2. Materials and Methods

### 2.1. Study Design

This study was designed as an assessor-blinded, two-arm, parallel-group randomized superiority trial with a pretest–posttest design. The trial was conducted to test whether adding DGTVA to a general exercise program would produce greater improvements in gait-related and functional outcomes than the general exercise program alone. Patients and the public were not involved in the design, conduct, reporting, or dissemination plans of this trial. No important changes to the trial protocol, prespecified outcomes, or analyses were made after trial commencement. Adverse events and unintended effects related to the intervention were monitored throughout the study period by the supervising physical therapist. No independent safety committee was established because this was a short-term, non-pharmacological exercise intervention. Participant safety was monitored during each intervention and assessment session by the supervising physical therapist, and any safety concerns were to be reported to the principal investigator and the Institutional Review Board according to institutional procedures. Adverse events were defined as any unfavorable physical or psychological symptom or incident occurring during or immediately after the intervention or assessment, including falls, near-falls, dizziness, musculoskeletal pain, excessive fatigue, cardiovascular symptoms, or other unexpected symptoms. If adverse events occurred, they were to be categorized by severity as mild, moderate, or severe/serious and by their relationship to the intervention. Mild events were defined as transient symptoms that did not require intervention or discontinuation; moderate events were defined as symptoms requiring rest, modification, or temporary cessation of the session; and severe or serious events were defined as events requiring medical evaluation, resulting in injury or hospitalization, or preventing continuation of the intervention. Although no formal trial-level stopping rules were planned, individual intervention sessions were to be stopped immediately if participants requested to stop or showed dizziness, chest pain, shortness of breath, excessive fatigue, musculoskeletal pain, loss of balance, near-fall, fall, or any other symptom judged unsafe by the supervising physical therapist. After completion of the baseline assessment, participants were randomly assigned in a 1:1 ratio to either the DGTVA group or the control group. The random allocation sequence was generated by an independent researcher using random allocation software version 2.0 (Mahmood Saghaei, Isfahan University of Medical Sciences, Isfahan, Iran, 2004) [16]. Simple randomization with a 1:1 allocation ratio was used. Allocation was concealed using sequentially numbered, opaque, sealed envelopes prepared by an independent researcher. Allocation was not disclosed until after completion of the baseline assessment. At that point, the independent researcher opened the next envelope in numerical sequence and disclosed the assigned group for intervention implementation. The outcome assessor was not involved in sequence generation, envelope preparation, or allocation disclosure and remained blinded to group allocation throughout the assessment process. All assessments of demographic characteristics, gait ability, balance, executive function, and fall efficacy were conducted by a single experienced physical therapist who was blinded to group allocation. Because participants and intervention therapists could not be blinded in this exercise-based intervention, participants were instructed before each post-intervention assessment not to disclose their group allocation, intervention content, training schedule, or exercise details to the assessor. The assessor was not involved in intervention delivery, did not observe training sessions, and conducted assessments in a separate assessment area. Group allocation was not recorded on assessment forms available to the assessor. Any accidental disclosure of group allocation during assessment was planned to be documented as an instance of unblinding. No instances of assessor unblinding were reported or documented during the study.

Participants were screened according to the inclusion and exclusion criteria. Because the study population consisted of older adults with MCI, participant autonomy and understanding were carefully considered during the consent process. Only individuals who were able to understand and follow the study procedures and verbal instructions were eligible for enrollment. Before enrollment, all potential participants and their legally authorized representatives received verbal and written explanations in plain language regarding the study purpose, random allocation process, intervention procedures, group differences, potential benefits and risks, time commitment, and right to withdraw. Written proxy consent was obtained from the participants’ legally authorized representatives, who were the guardians authorized to provide proxy consent under the IRB-approved consent procedure. The participants’ own understanding and willingness to participate were confirmed through a question-and-answer process before enrollment. Participants and their legally authorized representatives were informed that both groups would receive the conventional physical and cognitive training program, whereas only the DGTVA group would receive the additional visual adaptation-based dual-task gait training during the study period. They were also informed that refusal or withdrawal would not affect usual care or services at the adult day care center and that personal benefit from the intervention could not be guaranteed. The control group participated in a conventional physical and cognitive training program for 4 weeks (8 sessions in total, 60 min per session) and did not receive dual-task gait training or visual adaptation stimuli. The DGTVA group completed the same conventional program (8 sessions, 60 min per session) and additionally underwent a visual adaptation-based dual-task gait training program for 4 weeks (4 sessions per week, 60 min per session).

Outcome measures included demographic characteristics, gait ability, balance, executive function, and fall efficacy. Post-intervention assessments were conducted 1 week after the end of the intervention following the same procedures used at baseline. All collected data were organized and analyzed according to predefined procedures. This study was approved by the Institutional Review Board of Sahmyook University (SYU 2024-10-003, 25 November 2024). The trial was registered with the Clinical Research Information Service (CRIS), Republic of Korea, under the registration number KCT0011516.

### 2.2. Participants

Participants were recruited from older adults aged 65 years and older attending community-based adult day care centers in South Korea. After administration of the Montreal Cognitive Assessment Korean version (MoCA-K), individuals whose scores, interpreted according to the guidelines with consideration of educational level, were below 23 and thus classified in the MCI range were selected. Participants were included if they were able to perform basic activities of daily living, understand and follow the study procedures and verbal instructions, walk independently for at least 10 m without an assistive device, and complete the assessments with corrected vision and hearing. Exclusion criteria were neurological or musculoskeletal disorders that could substantially affect gait and balance performance, severe visual or hearing impairment that interfered with assessment, a history of lower-extremity surgery or fracture within the previous 6 months, and psychiatric problems such as moderate or greater depression or anxiety that could affect cognitive or motor performance. Individuals were also excluded when it was difficult to ensure the safety of study participation or the consistency of assessment performance.

During recruitment and allocation, 48 individuals were assessed for eligibility. Of these, 8 were excluded because their cognitive assessment results did not meet the selection criteria. Ultimately, 40 participants were included in the study and randomly assigned to the DGTVA group (*n* = 20) or the control group (*n* = 20). No participants dropped out during follow-up, and data from all 40 participants were included in the final analysis.

The MoCA-K was used to screen for MCI. The MoCA is a representative screening tool that assesses multiple cognitive domains, including attention, memory, and executive function, and has been reported to have high reliability and validity [17]. The assessment takes approximately 10 min to complete. In this study, MoCA-K scores were interpreted with consideration of participants’ educational level, and a score below 23 was used as the criterion for classification in the MCI range. The MoCA has been reported to show a sensitivity of 0.90 and a specificity of 0.87 for detecting MCI [17].

### 2.3. Randomization and Blinding

After completion of the baseline assessment, participants were randomly assigned in a 1:1 ratio to either the DGTVA group or the control group using a computer-based randomization program. All assessments were performed by an experienced physical therapist, and the evaluator was blinded to group allocation. Assessments of demographic characteristics, gait ability, balance, executive function, and fall efficacy were conducted by a single blinded assessor. Baseline gait and balance variables were additionally summarized to provide a clearer functional profile of the study population before intervention.

### 2.4. Intervention

#### 2.4.1. Dual-Task Gait Training with Visual Adaptation (DGTVA)

The DGTVA program was conducted using a wireless light system (Witty SEM, Microgate, Bolzano, Italy, 2017). The training was performed four times per week for 4 weeks, with each session lasting 60 min and consisting of a 10-min warm-up, 40-min main exercise period, and 10-min cool-down. The main exercise was designed as a dual-task-based training program in which participants responded to visual stimuli while walking and simultaneously performed cognitive tasks. It was structured to induce cognitive–motor interaction involving visual information processing and executive function. All training sessions were conducted under the supervision of an experienced physical therapist.

The difficulty of the DGTVA tasks was divided into five levels. The initial stage focused on simple selective-response tasks requiring participants to respond to specific color stimuli, thereby demanding visual attention and response selection. Thereafter, the difficulty was progressively increased to include response speed, inhibitory control, task switching, and working memory. Level 1 required responses to a green light and focused on visual attention. Level 2 required responses to a red light and emphasized rapid and accurate responses. Level 3 required responses to lights of the same color and involved visual pattern recognition and inhibitory control. Level 4 required responses to lights of the same shape and demanded inhibitory control and task switching. Level 5 required participants to remember the order of numbers and respond accordingly, thereby requiring integrated performance of working memory and executive function.

**Figure 1:**
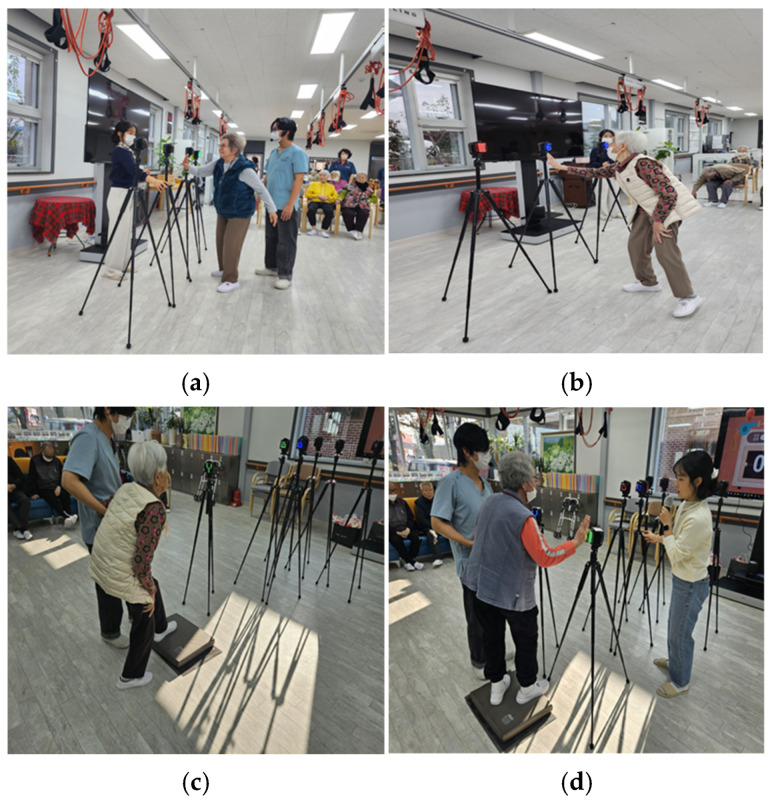
Weekly progression of DGTVA training across the four-week intervention: (**a**) Week 1, training with four lights and no obstacles; (**b**) Week 2, training with four lights and no obstacles; (**c**) Week 3, training with six lights and predictable obstacles; and (**d**) Week 4, training with eight lights, predictable obstacles, and unpredictable environmental stimuli.

In addition to the stepwise increase in task difficulty, the walking environment was also progressively modified according to the training week. During the first 2 weeks, training was performed using four lights and without obstacles so that participants could focus on adaptation to visual stimuli and dual-task performance. In week 3, the number of lights was increased to six, and predictable obstacles (e.g., hurdles and balance pads) were added, requiring planned avoidance strategies and balance control. In week 4, the number of lights was increased to eight, and both predictable obstacles and unpredictable environmental stimuli (e.g., sudden auditory stimuli) were applied, requiring real-time adaptation and cognitive flexibility in a complex environment. This structure was applied to gradually increase the executive demands and environmental adaptability required during walking (Figure 1).

#### 2.4.2. General Program

The general program consisted of physical and cognitive training routinely provided at the adult day care center. During the study period, this program was administered for a total of eight sessions, with each session lasting 60 min, and all sessions were conducted under the supervision of an experienced physical therapist. Each session consisted of physical exercise and cognitive exercise. The physical exercise component consisted primarily of non-ambulatory upper-extremity and coordination activities, such as sling exercises and cup-tapping exercises, to promote upper-extremity strength, flexibility, and wrist coordination. The general program did not include overground walking practice, obstacle negotiation, gait-specific training, dual-task gait training, or visual adaptation stimuli. The cognitive exercise component included tasks such as coloring activities to improve visual attention and coordination.

The control group participated in the general program, consisting of physical and cognitive training, for a total of eight sessions over 4 weeks, with each session lasting 60 min, and did not receive dual-task gait training or visual adaptation stimuli. The DGTVA group participated in the same general program for a total of eight sessions, with each session lasting 60 min, and additionally received the visual adaptation-based dual-task gait training program four times per week for 4 weeks, with each session lasting 60 min. Accordingly, the control group received 120 min/week of supervised conventional physical and cognitive training, whereas the DGTVA group received 120 min/week of the same conventional program plus 240 min/week of supervised visual adaptation-based dual-task gait training. This supervised intervention time included warm-up, cool-down, rest periods, safety monitoring, cognitive–motor task practice, visual stimulus–response practice, and obstacle negotiation tasks, and should therefore be interpreted as therapeutic training exposure rather than as an equivalent amount of continuous moderate-to-vigorous aerobic physical activity.

### 2.5. Outcome Measures

#### 2.5.1. Gait Ability Assessment

Gait ability was assessed using the optical sensor-based OptoGait system (OptoGait, Microgate, Bolzano, Italy, 2017). OptoGait is an optical sensor-based system that calculates spatiotemporal locomotor variables based on gait events and foot contact timing, and its test–retest reliability has been reported for spatiotemporal locomotor parameters [18]. The assessment was conducted on an indoor 10-m straight walkway with minimal external distractions, and data from the steady-state walking section, excluding the acceleration and deceleration phases, were used for analysis. Participants were instructed to walk at their preferred walking speed.

The basic walking and predictable obstacle walking conditions were each measured three times, and the mean value for each condition was used for analysis. In contrast, the unpredictable obstacle walking condition was not established to obtain a repeated mean value but to evaluate the initial gait response to a sudden stimulus. To minimize adaptation or familiarization due to repeated exposure and to capture the response to an unpredictable obstacle as much as possible, only the value from the single trial in which the stimulus was actually presented at each assessment time point was used for analysis [19,20]. Gait variables were classified and analyzed as spatiotemporal gait variables and adaptive gait variables.

- Spatiotemporal Gait Parameters

**Figure 2:**
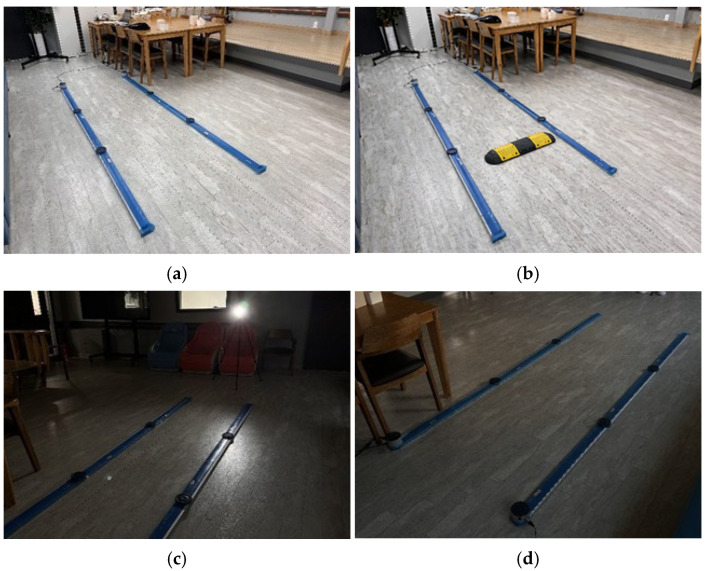
Gait ability under four walking conditions: (**a**) baseline walking; (**b**) predictable obstacle walking; (**c**) unpredictable obstacle walking under the light condition; and (**d**) unpredictable obstacle walking under the noise condition.

Spatiotemporal gait parameters were calculated under the conditions of Baseline walking, predictable obstacle walking, unpredictable obstacle walking (light), and unpredictable obstacle walking (noise) (Figure 2). The analyzed variables included step length, stride length, step time, stride time, cadence, total double support, and gait speed. These variables are representative indicators commonly used to evaluate gait efficiency and stability in older adults [21,22].

- Adaptive Gait Parameters

Adaptive gait assessment was performed under the conditions of predictable obstacle walking, unpredictable obstacle walking (light), and unpredictable obstacle walking (noise). To evaluate the dynamic stability of gait, step time variability, step length variability, step length symmetry, and total double support were calculated. Gait variability was calculated using the coefficient of variation (CV) [23].

The obstacle negotiation process was analyzed in three phases. The approach phase was defined as the preceding walking section before reaching the obstacle and included approximately 3–4 steps before the obstacle. The crossing phase was defined as the interval from the moment the leading foot crossed the obstacle to the moment the trailing foot crossed it. The post-crossing phase was defined as the approximately three-step section immediately after obstacle crossing. Among the adaptive gait variables, values for the predictable obstacle walking condition were calculated as the mean of three repeated trials, whereas values for the unpredictable obstacle walking conditions (light and noise) were derived from the single trial in which the stimulus was actually presented.

#### 2.5.2. Balance

Balance ability was assessed using the Berg Balance Scale (BBS). The BBS is a widely used tool for evaluating functional balance in older adults and consists of 14 items, each scored from 0 to 4, with a maximum total score of 56. In this study, the assessment was performed by an experienced physical therapist in a quiet and safe indoor environment according to standardized procedures. To maintain consistency, the same assessor conducted the evaluations before and after the intervention, and the assessor was blinded to group allocation. The assessment results were analyzed using the total score. The BBS is a widely used functional balance measure in older adults and has been used to predict fall probability in community-dwelling older adults [24]. Although ceiling effects have been reported when the BBS is used in higher-functioning community-dwelling older adults, it remains a clinically feasible and widely used measure of functional balance in older adult populations [25].

#### 2.5.3. Executive Function

- Stroop Test

Inhibitory control was assessed using the Korean Color-Word Stroop Test (K-CWST) [26]. The assessment was conducted by an experienced physical therapist under the same environmental conditions, and participants were instructed to respond to the presented stimuli as quickly and accurately as possible. In this study, the color naming condition was used, and completion time (s) and accuracy were used as the analysis variables.

- Trail Making Test

Cognitive flexibility and task-switching ability were assessed using the Korean Trail Making Test for the Elderly (K-TMT-e) [27]. The test consists of Part A and Part B, which assess visual scanning and attention, and task switching ability, respectively. In this study, the completion times for Part A and Part B were used as the analysis variables.

#### 2.5.4. Fall Efficacy

Fall efficacy was assessed using the Falls Efficacy Scale–International (FES-I). The FES-I is a tool used to assess fear of falling, concern about falling, and confidence in performing daily activities and, compared with the original FES, includes a wider range of daily and environmental situations, allowing more sensitive assessment of fall-related concern [28]. Concern about falling is clinically relevant because it is associated with future fall risk and is recommended as part of multifactorial fall-risk assessment in older adults [29]. The scale consists of 16 items, each scored from 1 to 4, with a total score ranging from 16 to 64. Higher scores indicate greater fear of falling and lower confidence.

In this study, the assessment was conducted by an experienced physical therapist in a separate room using a one-to-one interview format. To ensure consistency, the same assessor administered the test before and after the intervention, and the total score was used for the analysis. The FES-I has been reported to show high interrater reliability (r = 0.96) and intrarater reliability (r = 0.95) in older adults [30].

### 2.6. Statistical Analysis

The sample size was calculated using G*Power software (G*Power 3.1.9.7, Heinrich Heine Universität Düsseldorf, Düsseldorf, Germany, 2020). Because the primary analysis of this study involved repeated measures analysis of variance for group (2 levels) and time (2 levels), the F tests repeated measures within–between interaction model was applied. This study aimed to evaluate the effects of dual-tasking on gait speed in community-dwelling older adults, and a medium effect size (f = 0.25) was set based on a previous meta-analysis reporting that cognitive dual-tasking significantly reduced gait speed in community-dwelling older adults [31]. Using a significance level of 0.05, statistical power of 0.80, a correlation coefficient between repeated measures (ρ = 0.5), and an expected dropout rate of 10%, the required minimum sample size was calculated as 18 participants per group. No interim analyses or stopping guidelines were planned.

All statistical analyses were performed using IBM SPSS Statistics version 29.0 (IBM Corp., Armonk, NY, USA). All continuous variables were presented as mean ± standard deviation. The normality of continuous variables was examined using the Shapiro–Wilk test, and between-group homogeneity of participants’ general characteristics was assessed using the independent t test and the chi-square test.

Spatiotemporal gait variables were analyzed separately according to the four walking conditions (basic walking, predictable obstacle walking, unpredictable obstacle walking [light], and unpredictable obstacle walking [noise]). For each condition, a two-way mixed repeated measures analysis of variance (ANOVA) was performed, with group (2 levels: DGTVAG, CG) as the between-subjects factor and time (2 levels: pre, post) as the within-subjects factor.

Adaptive gait variables were also analyzed separately by walking condition. For each of the predictable obstacle walking, unpredictable obstacle walking (light), and unpredictable obstacle walking (noise) conditions, the walking task was divided into the approach phase, crossing phase, and post-crossing phase. A two-way mixed repeated-measures ANOVA was then performed for each dependent variable in each phase, with group (2 levels: DGTVAG, CG) and time (2 levels: pre, post) as factors.

The balance measure, the BBS, the executive function measures, including Stroop test accuracy and completion time and Trail Making Test Part A and Part B, and the fall efficacy measure, the FES-I, were likewise analyzed using two-way mixed repeated-measures ANOVA with group (2 levels: DGTVAG, CG) and time (2 levels: pre, post) as factors.

The level of statistical significance was set at α = 0.05, and effect sizes were presented as partial eta squared (*ηp*^2^). Because multiple interrelated gait, balance, executive function, and fall efficacy outcomes were analyzed, a global Bonferroni correction across all outcomes was not applied. Therefore, *p* values should be interpreted cautiously in light of multiple comparisons, and effect sizes were considered alongside statistical significance when interpreting the results. Participants were individually randomized, and no cluster randomization by center, therapist, or training group was used; therefore, cluster-adjusted analyses were not performed. No outcome data were missing; therefore, all randomized participants were included in the analyses according to their allocated groups.

## 3. Results

**Figure 3:**
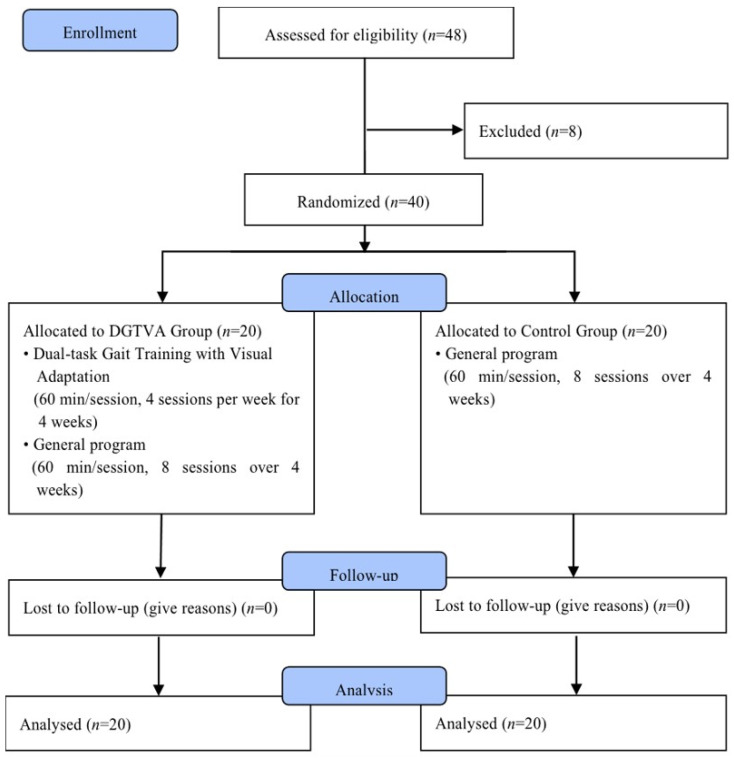
Participant flow through the trial.

Participants were recruited between November and December 2024, and post-intervention assessments were completed in February 2025. A total of 48 participants were assessed for eligibility. Of these, 8 were excluded because their cognitive assessment results did not meet the selection criteria. Ultimately, 40 participants were included in the study and randomly assigned to the DGTVA group (*n* = 20) or the control group (*n* = 20). No participants were lost to follow-up, and data from all 40 participants were included in the final analysis. The trial ended as planned after all participants completed the intervention and post-intervention assessments. No adverse events or unintended effects related to the intervention were reported or observed in either group during the study period. Therefore, the number of adverse events was 0 in both groups, and no events required severity or relatedness categorization. The flow of participants through the trial is shown in Figure 3.

### 3.1. Participant Flow and Baseline Characteristics

A total of 40 participants were included in this study and randomly assigned to the dual-task gait training with visual adaptation group (DGTVAG, *n* = 20) and the control group (CG, *n* = 20). The baseline demographic and clinical characteristics of the participants are presented in Table 1. No between-group differences were observed at baseline for sex, age, education level, MoCA-K, height, weight, BMI, or fall history (all *p* > 0.05). Fall history was reported by 3 participants in the DGTVAG (15.0%) and 2 participants in the CG (10.0%).

### 3.2. Primary Gait Outcomes

#### 3.2.1. Spatiotemporal Gait Parameters

Spatiotemporal gait parameters were evaluated under four walking conditions: baseline walking, predictable obstacle walking, unpredictable obstacle walking with light, and unpredictable obstacle walking with noise.

- Baseline Walking Condition

Significant main effects of time were observed for all spatiotemporal gait parameters, including step length, stride length, step time, stride time, cadence, total double support (%), and speed (all *p* < 0.05) (Table 2). Significant time × group interactions were observed for step length (F = 10.306, *p* = 0.003, *ηp*^2^ = 0.213), stride length (F = 62.677, *p* < 0.001, *ηp*^2^ = 0.623), stride time (F = 22.023, *p* < 0.001, *ηp*^2^ = 0.367), cadence (F = 5.116, *p* = 0.030, *ηp*^2^ = 0.119), and speed (F = 24.730, *p* < 0.001, *ηp*^2^ = 0.394). Specifically, the DGTVAG showed greater improvements than the CG, with step length increasing from 61.29 ± 5.20 cm to 68.40 ± 8.45 cm, stride length from 122.57 ± 4.48 cm to 136.79 ± 6.70 cm, cadence from 98.18 ± 6.94 steps/min to 113.34 ± 10.87 steps/min, and speed from 1.00 ± 0.09 m/s to 1.28 ± 0.12 m/s. In contrast, the CG showed only modest changes. Significant group effects were observed for step length (F = 10.438, *p* = 0.003, *ηp*^2^ = 0.215), stride length (F = 57.315, *p* < 0.001, *ηp*^2^ = 0.601), stride time (F = 7.614, *p* = 0.009, *ηp*^2^ = 0.167), and speed (F = 20.631, *p* < 0.001, *ηp*^2^ = 0.352). Step time and total double support (%) showed significant time effects only, without significant interaction or group effects (Table 2).

- Predictable Obstacle Walking Condition

Significant main effects of time were observed for all spatiotemporal gait parameters (all *p* < 0.05) (Table 3). Significant time × group interactions were observed for step length (F = 6.378, *p* = 0.016, *ηp*^2^ = 0.144), stride length (F = 34.262, *p* < 0.001, *ηp*^2^ = 0.474), step time (F = 14.287, *p* = 0.001, *ηp*^2^ = 0.273), stride time (F = 70.523, *p* < 0.001, *ηp*^2^ = 0.650), cadence (F = 14.168, *p* = 0.001, *ηp*^2^ = 0.272), and speed (F = 11.730, *p* = 0.001, *ηp*^2^ = 0.236). The DGTVAG showed greater improvements than the CG, with increased step length, stride length, cadence, and speed, as well as decreased step time and stride time. Significant group effects were observed for stride length (F = 17.480, *p* < 0.001, *ηp*^2^ = 0.315), step time (F = 7.340, *p* = 0.010, *ηp*^2^ = 0.162), stride time (F = 56.169, *p* < 0.001, *ηp*^2^ = 0.596), cadence (F = 12.080, *p* = 0.001, *ηp*^2^ = 0.241), and speed (F = 5.178, *p* = 0.029, *ηp*^2^ = 0.120). Total double support (%) showed a significant time effect only (F = 5.202, *p* = 0.028, *ηp*^2^ = 0.120), with no significant interaction or group effect (Table 3).

- Unpredictable Obstacle Walking Condition (Light)

Significant time × group interactions were observed for step length (F = 10.838, *p* = 0.002, *ηp*^2^ = 0.222), stride length (F = 53.730, *p* < 0.001, *ηp*^2^ = 0.586), and total double support (%) (F = 7.416, *p* = 0.010, *ηp*^2^ = 0.163) (Table 4). Step length increased in the DGTVAG (60.80 ± 5.44 cm to 64.36 ± 5.45 cm) but decreased in the CG (60.87 ± 5.74 cm to 58.34 ± 3.10 cm). Similarly, stride length increased in the DGTVAG (121.61 ± 3.93 cm to 128.72 ± 4.64 cm) but decreased in the CG (121.73 ± 5.26 cm to 116.69 ± 3.10 cm). Total double support (%) decreased in the DGTVAG (31.41 ± 3.38% to 28.06 ± 4.15%) but changed minimally in the CG (30.13 ± 5.23% to 30.45 ± 6.04%). Significant group effects were observed for step length (F = 5.225, *p* = 0.028, *ηp*^2^ = 0.121) and stride length (F = 30.271, *p* < 0.001, *ηp*^2^ = 0.443). Significant time effects were observed for stride time (F = 5.199, *p* = 0.028, *ηp*^2^ = 0.120) and total double support (%) (F = 5.084, *p* = 0.030, *ηp*^2^ = 0.118). No significant effects were found for step time, cadence, or speed (all *p* > 0.05) (Table 4).

- Unpredictable Obstacle Walking Condition (Noise)

Significant time × group interactions were observed for step length (F = 4.510, *p* = 0.040, *ηp*^2^ = 0.106) and stride length (F = 43.926, *p* < 0.001, *ηp*^2^ = 0.536) (Table 5). Step length increased in the DGTVAG (61.18 ± 8.10 cm to 66.52 ± 5.60 cm), whereas the CG showed little change (61.36 ± 8.44 cm to 60.54 ± 5.50 cm). Stride length increased in the DGTVAG (122.36 ± 5.70 cm to 133.04 ± 4.37 cm) but changed minimally in the CG (122.72 ± 7.10 cm to 121.08 ± 3.50 cm). A significant group effect was observed for stride length (F = 16.907, *p* < 0.001, *ηp*^2^ = 0.308). Significant time effects were found for stride length (F = 23.624, *p* < 0.001, *ηp*^2^ = 0.383), step time (F = 4.277, *p* = 0.045, *ηp*^2^ = 0.101), stride time (F = 18.405, *p* < 0.001, *ηp*^2^ = 0.326), cadence (F = 5.430, *p* = 0.025, *ηp*^2^ = 0.125), and speed (F = 5.685, *p* = 0.022, *ηp*^2^ = 0.130). No significant effects were observed for total double support (%) (all *p* > 0.05) (Table 5).

#### 3.2.2. Adaptive Gait Parameters

Adaptive gait parameters were evaluated under predictable and unpredictable obstacle walking conditions and were categorized into the approach, crossing, and post-crossing phases.

**Predictable Obstacle Condition.** In the approach phase, significant time × group interactions were observed for step time variability (F = 23.054, *p* < 0.001, *ηp*^2^ = 0.378) and step length variability (F = 8.588, *p* = 0.006, *ηp*^2^ = 0.184) (Table 6). Step time variability decreased in the DGTVAG (5.78 ± 1.40 to 4.22 ± 0.98) but increased in the CG (5.48 ± 1.28 to 5.85 ± 1.30). Step length variability also decreased to a greater extent in the DGTVAG (5.21 ± 1.62 to 3.51 ± 0.63) than in the CG (6.17 ± 1.43 to 5.56 ± 1.53). A significant group effect was observed for step length variability in the approach phase (F = 16.989, *p* < 0.001, *ηp*^2^ = 0.309). In the crossing phase, a significant time × group interaction was observed for total double support (%) (F = 18.028, *p* < 0.001, *ηp*^2^ = 0.322), along with a significant group effect (F = 6.853, *p* = 0.013, *ηp*^2^ = 0.153). Total double support (%) decreased in the DGTVAG (29.56 ± 3.59% to 26.21 ± 5.44%) but increased in the CG (29.96 ± 5.15% to 32.62 ± 4.74%). Significant time effects were also observed for step time variability in the approach and post-crossing phases, step length variability in the approach and post-crossing phases, and step length symmetry in the approach, crossing, and post-crossing phases (all *p* < 0.05). No other significant interaction or group effects were identified (Table 6).

**Unpredictable Obstacle Condition (Light).** Under the light condition, significant interaction and group effects were limited overall. A significant time × group interaction was found only for total double support (%) in the approach phase (F = 6.060, *p* = 0.018, *ηp*^2^ = 0.137) (Table 7). Specifically, total double support (%) decreased in the DGTVAG (32.10 ± 4.90% to 28.92 ± 3.30%) but increased in the CG (29.96 ± 4.57% to 32.33 ± 3.86%). Significant time effects were observed for post-crossing step time variability (F = 6.800, *p* = 0.013, *ηp*^2^ = 0.152), approach phase step length variability (F = 9.547, *p* = 0.004, *ηp*^2^ = 0.201), and step length symmetry in the approach (F = 4.991, *p* = 0.031, *ηp*^2^ = 0.116) and post-crossing phases (F = 5.061, *p* = 0.030, *ηp*^2^ = 0.118). No significant group effects were observed for any adaptive gait variable under this condition (Table 7).

**Unpredictable Obstacle Condition (Noise).** Under the noise condition, significant time × group interactions were observed for step time variability in the approach phase (F = 7.435, *p* = 0.010, *ηp*^2^ = 0.164) and post-crossing phase (F = 19.957, *p* < 0.001, *ηp*^2^ = 0.344), step length variability in the post-crossing phase (F = 25.059, *p* < 0.001, *ηp*^2^ = 0.397), step length symmetry in the crossing phase (F = 10.372, *p* = 0.003, *ηp*^2^ = 0.214) and post-crossing phase (F = 6.413, *p* = 0.016, *ηp*^2^ = 0.144), and total double support (%) in the crossing phase (F = 6.467, *p* = 0.015, *ηp*^2^ = 0.145) (Table 8). Significant group effects were observed for step time variability in the approach phase (F = 4.533, *p* = 0.040, *ηp*^2^ = 0.107) and post-crossing phase (F = 4.653, *p* = 0.037, *ηp*^2^ = 0.109), as well as for step length variability in the post-crossing phase (F = 8.966, *p* = 0.005, *ηp*^2^ = 0.191). Specifically, approach-phase step time variability decreased in the DGTVAG (5.44 ± 1.35 to 4.68 ± 1.17) but increased in the CG (5.64 ± 1.31 to 6.05 ± 1.54). Post-crossing step time variability showed a greater reduction in the DGTVAG (8.26 ± 1.35 to 4.28 ± 0.64), whereas the reduction in the CG was smaller (7.79 ± 1.31 to 6.93 ± 3.33). Post-crossing step length variability decreased in the DGTVAG (7.97 ± 1.54 to 4.76 ± 0.83) but slightly increased in the CG (7.77 ± 1.35 to 8.01 ± 3.19). During the crossing phase, step length symmetry decreased in the DGTVAG (15.61 ± 10.84 to 8.38 ± 3.52) but increased in the CG (12.22 ± 10.89 to 15.19 ± 9.92). A similar pattern was observed in the post-crossing phase (DGTVAG: 13.04 ± 3.64 to 9.83 ± 3.06; CG: 11.42 ± 2.96 to 12.50 ± 7.78). For total double support (%) during the crossing phase, the DGTVAG decreased (31.51 ± 5.94% to 27.63 ± 5.21%), whereas the CG increased (29.63 ± 7.43% to 30.98 ± 7.14%). In addition, significant time effects were observed for post-crossing step time variability (F = 47.800, *p* < 0.001, *ηp*^2^ = 0.557), crossing-phase step length variability (F = 8.083, *p* = 0.007, *ηp*^2^ = 0.175), post-crossing step length variability (F = 18.744, *p* < 0.001, *ηp*^2^ = 0.330), approach-phase step length symmetry (F = 48.519, *p* < 0.001, *ηp*^2^ = 0.561), and approach-phase total double support (%) (F = 7.749, *p* = 0.008, *ηp*^2^ = 0.169) (Table 8).

### 3.3. Secondary Outcomes

#### 3.3.1. Balance Outcomes 

A significant main effect of time was observed for the Berg Balance Scale (BBS) scores (F = 313.771, *p* < 0.001, *ηp*^2^ = 0.892). In addition, the time × group interaction was significant (F = 174.197, *p* < 0.001, *ηp*^2^ = 0.821), indicating that the pattern of change over time differed between the two groups. However, the main effect of group was not significant (F = 3.398, *p* = 0.073, *ηp*^2^ = 0.082). The DGTVAG showed a statistically significant increase in BBS scores from pre- to post-intervention (45.55 ± 2.33 to 50.00 ± 2.15; mean change, +4.45 points), whereas the CG showed only a slight increase (46.05 ± 2.21 to 46.70 ± 2.99; mean change, +0.65 points) (Table 9).

#### 3.3.2. Executive Function Outcomes

For Stroop test accuracy, significant main effects of time (F = 224.234, *p* < 0.001, *ηp*^2^ = 0.855) and group (F = 8.307, *p* = 0.006, *ηp*^2^ = 0.179) were observed, and the time × group interaction was also significant (F = 134.576, *p* < 0.001, *ηp*^2^ = 0.780). The DGTVAG showed a greater increase in accuracy from pre- to post-intervention (79.17 ± 3.61% to 84.42 ± 3.35%) than the CG (78.33 ± 3.63% to 79.00 ± 3.35%). For Stroop test completion time, a significant main effect of time (F = 275.752, *p* < 0.001, *ηp*^2^ = 0.879) and a significant time × group interaction (F = 290.376, *p* < 0.001, *ηp*^2^ = 0.884) were observed, whereas the main effect of group was not significant (F = 2.090, *p* = 0.156, *ηp*^2^ = 0.052). The DGTVAG showed a marked reduction in completion time (69.40 ± 6.73 s to 58.95 ± 4.54 s), whereas the CG showed little change (66.35 ± 4.09 s to 66.49 ± 4.21 s) (Table 10).

For the Trail Making Test Part A, a significant main effect of time (F = 173.281, *p* < 0.001, *ηp*^2^ = 0.820) and a significant time × group interaction (F = 285.095, *p* < 0.001, *ηp*^2^ = 0.882) were observed, but the main effect of group was not significant (F = 2.206, *p* = 0.146, *ηp*^2^ = 0.055). The DGTVAG showed a clear reduction in completion time (47.81 ± 5.32 s to 41.30 ± 3.91 s), whereas the CG showed minimal change (46.06 ± 3.37 s to 46.86 ± 3.59 s). For the Trail Making Test Part B significant main effects of time (F = 509.802, *p* < 0.001, *ηp*^2^ = 0.931) and group (F = 15.971, *p* < 0.001, *ηp*^2^ = 0.296) were found, and the time × group interaction was also significant (F = 586.708, *p* < 0.001, *ηp*^2^ = 0.939). The DGTVAG showed a marked reduction in completion time (98.87 ± 7.04 s to 87.82 ± 5.85 s), whereas the CG showed almost no change (99.82 ± 3.69 s to 100.21 ± 4.04 s) (Table 10).

#### 3.3.3. Fall Efficacy Outcomes

A significant main effect of time was observed for FES-I scores (F = 509.474, *p* < 0.001, *ηp*^2^ = 0.931). In addition, the time × group interaction was significant (F = 396.649, *p* < 0.001, *ηp*^2^ = 0.913), indicating that the pattern of change over time differed between the two groups. However, the main effect of group was not significant (F = 0.468, *p* = 0.498, *ηp*^2^ = 0.012). The DGTVAG showed a statistically significant decrease in FES-I scores from pre- to post-intervention (28.55 ± 2.33 to 23.75 ± 2.31; mean change, −4.80 points), whereas the CG showed little change (26.95 ± 3.47 to 26.65 ± 3.72; mean change, −0.30 points) (Table 11).

## 4. Discussion

### 4.1. Main Findings

This study examined the effects of DGTVA on gait ability, balance, executive function, and fall efficacy in older adults with mild cognitive impairment. The DGTVA group demonstrated greater improvements than the control group in several spatiotemporal gait parameters during both usual walking and predictable obstacle negotiation. In unpredictable conditions, by contrast, the training effect was reflected not in a generalized change across mean gait measures, but in more selective patterns of adaptation. In addition, statistically significant improvements were observed in balance and executive function, accompanied by a reduction in fall-related concern.

From this perspective, a closer examination of the gait outcomes suggests that the effects of DGTVA were consistently observed in both usual walking and predictable obstacle negotiation, with the latter showing improvements in temporal as well as spatial parameters. During usual walking, the DGTVA group demonstrated increases in step length from 61.29 to 68.40 cm, stride length from 122.57 to 136.79 cm, and gait speed from 1.00 to 1.28 m/s. Similarly, during predictable obstacle negotiation, step length increased from 61.41 to 68.14 cm, stride length from 122.82 to 136.27 cm, and gait speed from 1.02 to 1.27 m/s, with significant interaction effects also observed for cadence and step/stride time. The baseline gait speed should be considered when interpreting these findings. The DGTVA group walked at approximately 1.00 m/s during level walking at baseline, suggesting that participants had relatively preserved mobility rather than severe gait slowing. Previous studies on walking speed decline, normative walking speed, and prognosis have suggested that usual gait speed around or above 1.0 m/s generally reflects relatively preserved mobility in older adults, whereas slower speeds, particularly below approximately 0.8 m/s, are more strongly associated with functional vulnerability and poorer outcomes [6,32,33]. Therefore, the observed improvement from 1.00 to 1.28 m/s should be interpreted not as recovery from marked gait impairment, but as an enhancement of gait efficiency in older adults with MCI who already demonstrated reasonably good baseline walking capacity. This point also highlights why the adaptive gait outcomes during obstacle negotiation are particularly important: the intervention effects were reflected not only in faster walking, but also in more coordinated spatial, temporal, and phase-specific gait regulation during complex walking conditions. This pattern suggests that DGTVA did not merely increase walking speed, but facilitated a more efficient gait pattern through concurrent modulation of step length and gait rhythm. Of particular note, both spatial parameters (step length and stride length) and temporal parameters (cadence and step/stride time) improved during predictable obstacle negotiation. Gait is a complex task that integrates rhythm generation, balance control, and environmental adaptation [5], and predictable obstacle negotiation, unlike level walking, requires visual scanning during the approach phase, foot placement planning based on advance knowledge of obstacle location and crossing timing, and rhythmic adjustment during obstacle clearance [9]. These findings therefore suggest that DGTVA may have enhanced overall gait efficiency as well as anticipatory gait control in predictable obstacle contexts. In other words, the training appears to have improved the ability to plan and adjust gait strategy from the approach phase onward using advance visual information, which is consistent with the concomitant improvements observed across speed, spatial, and temporal gait measures.

By contrast, under unpredictable obstacle negotiation, the intervention effects were observed more selectively and only in specific gait variables. In the obstacle-walking condition with visual stimuli, significant time × group interactions were found for step length, stride length, and total double support, whereas in the obstacle-walking condition with auditory stimuli, significant time × group interactions were identified for step length and stride length. In the visual-stimulus condition, step length in the DGTVA group increased from 60.80 to 64.36 cm, whereas the control group showed a decrease from 60.87 to 58.34 cm. In the auditory-stimulus condition, stride length in the DGTVA group increased from 122.36 to 133.04 cm, whereas the control group showed little change, from 122.72 to 121.08 cm. This pattern suggests that, under unpredictable conditions, the effects of DGTVA were not consistently expressed across all mean spatiotemporal gait parameters, but were instead selectively captured in variables associated with reactive gait control, that is, the real-time adjustment of ongoing walking in response to sudden stimuli. Gait responses to unexpected environmental changes require rapid modification of the ongoing stepping plan and foot trajectory, and these demands are greater than those imposed by predictable walking conditions. Nevertheless, the DGTVA group demonstrated increases in step length and stride length, together with a concurrent change in total double support under the visual-stimulus condition, and maintained or improved step length and stride length under the auditory-stimulus condition. These findings suggest that even when unpredictable stimuli were presented, the DGTVA group did not adopt an overly conservative strategy of excessively shortening step length, but instead preserved forward progression. In other words, rather than responding with a passive avoidance strategy, they appeared to adapt in a way that allowed them to process stimulus information while maintaining basic locomotor efficiency. This interpretation is consistent with previous findings showing that dual-task interference on gait speed increases as both motor-task difficulty and cognitive-task difficulty rise, and that the combination of these two forms of difficulty may further amplify interference effects [10].

These intervention effects were more clearly evident in phase-specific adaptive gait variables than in general spatiotemporal gait variables. In particular, unlike the unpredictable obstacle-walking conditions in which visual or auditory stimuli were presented randomly, the predictable obstacle-walking condition may be interpreted as more directly reflecting changes in anticipatory gait regulation, because participants were able to approach the obstacle with prior knowledge of its location and the timing of crossing. Indeed, under the predictable condition, the DGTVA group showed a reduction in step time variability during the approach phase from 5.78 to 4.22 and a reduction in step length variability from 5.21 to 3.51. In the crossing phase, total double support percentage decreased from 29.56% to 26.21% in the DGTVA group, whereas it increased from 29.96% to 32.62% in the control group. These findings indicate that the DGTVA group regulated gait timing and foot placement more consistently during the approach phase before reaching the obstacle. In addition, the reduction in total double support during the crossing phase suggests that participants negotiated the obstacle while relying less on bilateral support at the moment of crossing. This interpretation is consistent with the findings of Yun and Park [9], who reported that as obstacle conditions become more complex, older adults may exhibit more pronounced adaptive changes during the pre-crossing phase than in actual crossing speed or clearance.

Phase-specific adaptive gait outcomes under the unpredictable obstacle-walking conditions showed distinct patterns according to stimulus modality. In the visual-stimulus condition, a significant time × group interaction was observed in total double support percentage during the approach phase, which may indicate a change in stability regulation during the preparatory period immediately before the onset of the unexpected visual stimulus. In contrast, in the auditory-stimulus condition, significant time × group interactions were found in step time variability during the approach phase; in step time variability and step length variability during the post-crossing phase; in step length symmetry during the crossing and post-crossing phases; and in total double support percentage during the crossing phase. Notably, in the DGTVA group, post-crossing step time variability decreased from 8.26 to 4.28, and post-crossing step length variability decreased from 7.97 to 4.76. These findings suggest that the effects of DGTVA were not confined to anticipatory regulation before obstacle approach, but may also have extended to the recovery and re-stabilization of gait rhythm and stepping patterns after the unexpected stimulus. This interpretation may also be indirectly linked to the view of Verghese et al. [22], who suggested that gait indices related to variability and rhythm may be more sensitive markers of future cognitive decline and dementia risk than single measures such as average gait speed.

The executive function findings provide important support for the mechanisms underlying the observed gait changes. In the present study, the DGTVA group showed an increase in Stroop accuracy from 79.17% to 84.42% and a decrease in Stroop completion time from 69.40 s to 58.95 s. Trail Making Test Part A decreased from 47.81 s to 41.30 s, and Trail Making Test Part B decreased from 98.87 s to 87.82 s. These findings are consistent with the design of DGTVA, which was intended to progressively challenge selective attention, processing speed, inhibitory control, and task switching during walking. In particular, the improvements in TMT-A and TMT-B suggest that DGTVA may have repeatedly stimulated visual scanning, cognitive flexibility, and set-shifting ability within a locomotor context. Kang and Baek [1] reported that individuals with MCI show more pronounced deficits not only in delayed recall but also in visual memory and frontal/executive function, and Yogev-Seligmann et al. [7] identified executive function and attention as key regulatory components of both usual and complex gait. Taken together, the concurrent improvements in multiple executive subdomains and gait performance suggest that DGTVA may not have enhanced cognition in isolation from gait, but rather strengthened the cognitive control processes required for obstacle negotiation.

Changes in balance ability and fall-related concern further support the functional relevance of the intervention effects, although they should be interpreted in light of the baseline scores and measurement characteristics. The BBS score in the DGTVA group increased from 45.55 to 50.00, whereas the control group showed only a minimal change from 46.05 to 46.70. Although baseline BBS scores indicated relatively preserved functional balance, this pattern suggests that DGTVA may have produced additional balance-related benefits beyond usual program participation. Nevertheless, because the BBS may show ceiling effects in community-dwelling older adults [25], the balance findings should be interpreted together with the gait and adaptive walking outcomes rather than in isolation. Similarly, FES-I scores decreased from 28.55 to 23.75 in the DGTVA group, whereas the control group showed little change from 26.95 to 26.65. Although participants had MCI, concern about falling remains a relevant construct in this population because MCI is associated not only with cognitive decline but also with gait, balance, and executive control impairments that may influence perceived safety during daily mobility [3,29]. Because the FES-I assesses concern about falling during daily activities rather than fear alone [28,30], this reduction may reflect improved perceived mobility confidence, particularly when accompanied by concurrent improvements in gait performance, balance, and executive function. However, because fall incidence was not prospectively monitored, these findings should be interpreted as evidence of improved fall-related confidence rather than direct evidence of fall reduction.

### 4.2. Comparison with Previous Literature

Previous dual-task gait studies involving older adults with MCI have been designed primarily in the context of screening and diagnosis. Wang et al. [2] reported that a dual-task gait assessment combining straight-path walking with an animal picture naming task could distinguish MCI with relatively good sensitivity; however, the main purpose of that study was to screen for possible MCI in community-dwelling older adults, and the outcome measure was limited to gait speed. In addition, the cognitive tasks commonly used in dual-task paradigms for MCI have mainly involved language processing or continuous cognitive tracking, such as serial subtraction, verbal fluency, and delayed recall (short-term memory tasks), whereas tasks requiring discrimination and decision-making have been relatively uncommon [2]. In contrast, the present study addressed dual-task gait not simply as a screening indicator but as a target of intervention, and incorporated obstacle negotiation into both training and assessment rather than limiting the task to straight-path walking. In particular, unobstructed walking, predictable obstacle walking, and unpredictable obstacle walking were organized in a stepwise manner to more directly address walking situations that require adaptation to real-world environments. Furthermore, by assessing not only gait speed but also spatiotemporal gait parameters, adaptive gait parameters, balance ability, executive function, and fall efficacy, this study examined dual-task gait in older adults with MCI from the perspective of cognitive–motor adaptive function in real-world environments.

Previous intervention studies provide partial support for the therapeutic potential of dual-task and obstacle-related training in older adults, but they differ from the present study in population, task structure, and outcome focus. A recent meta-analysis reported that dual-task training can improve cognitive and physical outcomes in older adults with MCI or dementia, although the included interventions were heterogeneous and were not specifically designed around visually guided obstacle negotiation [15]. Kuo et al. also showed that different dual-task training approaches may influence dual-task walking and brain activation in older adults with MCI [14]. In older adults without MCI, Hiyamizu et al. reported that dual-task balance training improved dual-task performance [12], and Yamada et al. showed that complex obstacle negotiation exercise could reduce falls in community-dwelling older adults aged 75 years and older [13]. The present study extends these findings by integrating visual adaptation, dual-task walking, and obstacle negotiation into a single gait-specific intervention and by evaluating both spatiotemporal and phase-specific adaptive gait outcomes.

### 4.3. Clinical Implications

In clinical terms, the observed improvements support the potential value of DGTVA as an add-on, task-specific cognitive–motor rehabilitation strategy for older adults with MCI. The intervention integrated visual adaptation, dual-task practice, obstacle negotiation, gait regulation, and postural stabilization, and therefore targeted the cognitive–motor adaptability required for complex community mobility. This is particularly relevant because older adults with MCI may show difficulty adapting gait to environmental demands even when usual walking ability is relatively preserved. Although the DGTVA group received more supervised gait-specific practice than the control group, the findings remain clinically meaningful as evidence that a structured multicomponent gait training package can improve gait performance, adaptive walking control, balance-related function, executive function, and fall-related confidence. Future dose-matched studies are needed to clarify the relative contribution of visual adaptation, dual-task training content, and training dose.

Clinically, DGTVA may be particularly useful for older adults with MCI who retain independent walking ability but remain vulnerable during complex walking situations, such as dual-task walking and obstacle negotiation. By repeatedly exposing participants to visual stimulus–response demands during gait, DGTVA may help improve the ability to maintain forward progression, regulate step timing and foot placement, and recover gait stability after unexpected environmental stimuli.

### 4.4. Mechanistic Interpretation

The beneficial effects of DGTVA may be explained by enhanced cognitive–motor integration during walking. The intervention required participants to process visual information, select relevant stimuli, inhibit inappropriate responses, and adapt their gait while negotiating environmental demands. Repeated exposure to these task requirements may have strengthened executive control and visual attentional processing, which in turn may have contributed to anticipatory gait adjustment under predictable obstacle conditions and reactive gait stabilization under unpredictable conditions. The concurrent improvements in Stroop performance, Trail Making Test performance, and adaptive gait measures support the interpretation that DGTVA influenced not only motor output but also the cognitive control processes underlying safe mobility. Because neurophysiological and detailed kinematic mechanisms were not directly measured, this mechanistic interpretation should be considered plausible and hypothesis-generating.

### 4.5. Limitations and Future Directions

Several considerations should be noted when interpreting the findings. First, the total supervised training dose and amount of gait-specific practice were not matched between the DGTVA and control groups. Therefore, the present findings should be interpreted as the effects of adding a structured DGTVA package to a conventional physical and cognitive training program. This does not diminish the clinical relevance of the findings, because DGTVA was intentionally designed as a multicomponent cognitive–motor gait training package integrating visual stimulus–response adaptation, dual-task practice, and obstacle negotiation. However, future dose-matched studies are needed to determine the specific contribution of visual adaptation beyond therapist contact time and gait-specific practice. Second, standardized habitual physical activity level was not assessed at baseline. Nevertheless, the two groups were comparable in demographic characteristics, cognitive status, baseline gait speed under level walking and obstacle negotiation conditions, and functional balance ability. In addition, the reported intervention dose should be interpreted as supervised therapeutic training exposure rather than as an equivalent amount of continuous moderate-to-vigorous aerobic physical activity, because each session included warm-up, cool-down, rest periods, safety monitoring, cognitive–motor task practice, visual stimulus–response practice, and obstacle negotiation tasks. Third, participant and intervention therapist blinding was not possible because the DGTVA group received additional gait-specific training with visible visual adaptation stimuli. To protect outcome assessor blinding, the assessor was separated from intervention delivery, assessments were conducted in a separate assessment area, group allocation was not recorded on assessor-accessible forms, and participants were instructed not to disclose their allocation or intervention content. No instances of assessor unblinding were reported or documented, although formal testing of blinding integrity was not performed. Fourth, the sample size was relatively small and participants were recruited from a single adult day care center, which may limit generalizability and precluded formal examination of center-level cluster effects. Fifth, multiple outcomes were analyzed across several walking conditions and obstacle negotiation phases, and no Bonferroni correction or other formal multiplicity adjustment was applied. Therefore, statistically significant findings, particularly those from secondary and phase-specific adaptive gait outcomes, should be interpreted with consideration of the potential risk of type I error. Future trials should prespecify a more limited set of primary outcomes and consider appropriate multiplicity adjustment or hierarchical testing procedures. Finally, because dual-task cost, cognitive task performance during walking, and neurophysiological indicators were not directly measured, the mechanisms underlying DGTVA cannot be definitively determined. Future studies should include larger multicenter samples, dose-matched active control groups with comparable therapist contact time and gait-specific practice, standardized physical activity assessment, long-term fall monitoring, and prespecified responder analyses based on MCID, MDC, or SEM thresholds. In addition, kinematic indicators such as toe clearance, trunk control, and gaze strategy, together with neurophysiological indicators such as prefrontal cortical activation, should be analyzed to clarify the mechanisms underlying adaptation to unpredictable environments. It will also be important to examine older adults with a wider range of functional levels, baseline fall-related concern, and fall-risk profiles.

## 5. Conclusions

In conclusion, dual-task gait training with visual adaptation produced statistically significant improvements in gait performance in older adults with mild cognitive impairment. The intervention was associated with clear gains in spatiotemporal gait parameters during level walking and predictable obstacle negotiation, as well as selective improvements in adaptive gait control under unpredictable obstacle conditions. In addition, balance and executive function improved, and concern about falling decreased after training. These findings suggest that gait training that integrates visual information processing, obstacle negotiation, and executive control may enhance the cognitive-motor adaptability required for safe mobility in daily life. Although the results should be interpreted cautiously because of the small single-center sample and the difference in total training dose, therapist contact time, and gait-specific practice between groups, the present study supports the potential clinical value of adding dual-task gait training with visual adaptation to a general exercise program as a task-specific rehabilitation strategy for older adults with mild cognitive impairment. Future studies should include larger samples and dose-matched active control groups with comparable total training dose, therapist contact time, and gait-specific practice. Longer-term follow-up is also needed to determine whether these benefits are sustained and whether they translate into actual reductions in falls and greater functional independence.

## Figures and Tables

**Table 1:** General characteristics of subjects.

	DGTVAG ^1^ (*n* = 20)	CG ^2^ (*n* = 20)	*χ*^2^/*t*(*p*)
Education level (years)	11.10 ± 1.41	11.55 ± 1.10	1.125 (0.267)
MoCA-K ^3^	22.15 ± 1.73	21.35 ± 1.67	1.486 (0.144)
Gender (male/female)	5/15	6/14	0.125 (0.723)
Age (year)	75.09 ± 6.32	73.70 ± 5.72	0.711 (0.481)
Height (cm)	158.32 ± 8.73	157.24 ± 10.92	0.335 (0.739)
Weight (kg)	62.25 ± 11.53	62.31 ± 6.73	0.020 (0.984)
BMI ^4^ (kg/m^2^)	25.23 ± 6.17	25.55 ± 4.59	0.184 (0.855)
Fall history (%)	15.00	10.00	1.000

^1^ dual-task gait training with visual adaptation group; ^2^ control group, ^3^ Montreal Cognitive Assessment-Korean version; ^4^ Body mass index. Values are expressed as mean ± standard deviation or %. Fall history refers to at least one fall within the previous 12 months. *p* values were calculated using the independent *t*-test, chi-square test, or Fisher’s exact test, as appropriate.

**Table 2:** Between-group comparison of spatiotemporal gait parameters in the baseline walking condition.

	DGTVAG ^1^ (*n* = 20)		CG ^2^ (*n* = 20)		Time (F, *p*, ${\eta p}^{2}$)	Time × Group (F, *p*, ${\eta p}^{2}$)	Group (F, *p*, ${\eta p}^{2}$)
	Pre	Post	Pre	Post			
Step length (cm)	61.29 ± 5.20	68.40 ± 8.45	60.99 ± 5.04	59.82 ± 4.28	5.279, 0.027, 0.122	10.306, 0.003, 0.213	10.438, 0.003, 0.215
Stride length (cm)	122.57 ± 4.48	136.79 ± 6.70	121.99 ± 4.19	119.63 ± 4.05	32.106, <0.001, 0.458	62.677, <0.001, 0.623	57.315, <0.001, 0.601
Step time (s)	0.61 ± 0.04	0.53 ± 0.05	0.61 ± 0.04	0.56 ± 0.02	57.531, <0.001, 0.602	3.738, 0.061, 0.090	1.341, 0.254, 0.034
Stride time (s)	1.23 ± 0.04	1.07 ± 0.04	1.22 ± 0.03	1.12 ± 0.02	338.951, <0.001, 0.899	22.023, <0.001, 0.367	7.614, 0.009, 0.167
Cadence (steps/min)	98.18 ± 6.94	113.34 ± 10.87	98.95 ± 6.67	107.01 ± 4.18	54.845, <0.001, 0.591	5.116, 0.030, 0.119	2.382, 0.131, 0.059
Total double support (%)	31.06 ± 3.88	26.21 ± 2.37	30.38 ± 3.75	28.32 ± 4.18	14.331, 0.001, 0.274	2.332, 0.135, 0.058	1.087, 0.304, 0.028
Speed (m/s)	1.00 ± 0.09	1.28 ± 0.12	1.01 ± 0.11	1.07 ± 0.07	58.344, <0.001, 0.606	24.730, <0.001, 0.394	20.631, <0.001, 0.352

^1^ dual-task gait training with visual adaptation group; ^2^ control group. Values are expressed as mean ± standard deviation.

**Table 3:** Between-group comparison of spatiotemporal gait parameters in the predictable obstacle walking condition.

	DGTVAG ^1^ (*n* = 20)		CG ^2^ (*n* = 20)		Time (F, *p*, ${\eta p}^{2}$)	Time × Group (F, *p*, ${\eta p}^{2}$)	Group (F, *p*, ${\eta p}^{2}$)
	Pre	Post	Pre	Post			
Step length (cm)	61.41 ± 5.25	68.14 ± 7.49	62.53 ± 6.99	61.99 ± 5.72	4.633, 0.038, 0.109	6.378, 0.016, 0.144	3.061, 0.088, 0.075
Stride length (cm)	122.82 ± 4.75	136.27 ± 5.91	125.05 ± 6.42	123.98 ± 4.56	24.888, <0.001, 0.396	34.262, <0.001, 0.474	17.480, <0.001, 0.315
Step time (s)	0.61 ± 0.04	0.54 ± 0.01	0.61 ± 0.04	0.58 ± 0.02	50.744, <0.001, 0.572	14.287, 0.001, 0.273	7.340, 0.010, 0.162
Stride time (s)	1.22 ± 0.04	1.07 ± 0.02	1.21 ± 0.02	1.16 ± 0.03	250.482, <0.001, 0.868	70.523, <0.001, 0.650	56.169, <0.001, 0.596
Cadence (steps/min)	99.47 ± 6.00	111.92 ± 3.17	99.65 ± 7.08	103.26 ± 4.31	46.758, <0.001, 0.552	14.168, 0.001, 0.272	12.080, 0.001, 0.241
Total double support (%)	31.50 ± 3.54	27.71 ± 2.65	30.87 ± 4.41	30.18 ± 4.71	5.202, 0.028, 0.120	2.504, 0.122, 0.062	1.502, 0.228, 0.038
Speed (m/s)	1.02 ± 0.07	1.27 ± 0.13	1.04 ± 0.26	1.07 ± 0.12	17.711, <0.001, 0.318	11.730, 0.001, 0.236	5.178, 0.029, 0.120

^1^ dual-task gait training with visual adaptation group; ^2^ control group. Values are expressed as mean ± standard deviation.

**Table 4:** Between-group comparison of spatiotemporal gait parameters in the unpredictable obstacle walking condition (light).

	DGTVAG ^1^ (*n* = 20)		CG ^2^ (*n* = 20)		Time (F, *p*, ${\eta p}^{2}$)	Time × Group (F, *p*, ${\eta p}^{2}$)	Group (F, *p*, ${\eta p}^{2}$)
	Pre	Post	Pre	Post			
Step length (cm)	60.80 ± 5.44	64.36 ± 5.45	60.87 ± 5.74	58.34 ± 3.10	0.313, 0.579, 0.008	10.838, 0.002, 0.222	5.225, 0.028, 0.121
Stride length (cm)	121.61 ± 3.93	128.72 ± 4.64	121.73 ± 5.26	116.69 ± 3.10	1.550, 0.221, 0.039	53.730, <0.001, 0.586	30.271, <0.001, 0.443
Step time (s)	0.62 ± 0.14	0.59 ± 0.10	0.64 ± 0.12	0.61 ± 0.15	0.941, 0.338, 0.024	0.005, 0.942, 0.000	0.642, 0.428, 0.017
Stride time (s)	1.24 ± 0.12	1.18 ± 0.08	1.28 ± 0.09	1.22 ± 0.14	5.199, 0.028, 0.120	0.030, 0.864, 0.001	3.517, 0.068, 0.085
Cadence (steps/min)	102.39 ± 24.52	104.42 ± 17.75	98.43 ± 17.62	104.59 ± 26.79	0.632, 0.431, 0.016	0.161, 0.690, 0.004	0.163, 0.688, 0.004
Total double support (%)	31.41 ± 3.38	28.06 ± 4.15	30.13 ± 5.23	30.45 ± 6.04	5.084, 0.030, 0.118	7.416, 0.010, 0.163	0.166, 0.686, 0.004
Speed (m/s)	1.05 ± 0.30	1.12 ± 0.20	1.00 ± 0.20	1.02 ± 0.27	0.694, 0.410, 0.018	0.216, 0.644, 0.006	1.868, 0.180, 0.047

^1^ dual-task gait training with visual adaptation group; ^2^ control group. Values are expressed as mean ± standard deviation.

**Table 5:** Between-group comparison of spatiotemporal gait parameters in the unpredictable obstacle walking condition (noise).

	DGTVAG ^1^ (*n* = 20)		CG ^2^ (*n* = 20)		Time (F, *p*, ${\eta p}^{2}$)	Time × Group (F, *p*, ${\eta p}^{2}$)	Group (F, *p*, ${\eta p}^{2}$)
	Pre	Post	Pre	Post			
Step length (cm)	61.18 ± 8.10	66.52 ± 5.60	61.36 ± 8.44	60.54 ± 5.50	2.425, 0.128, 0.060	4.510, 0.040, 0.106	2.946, 0.094, 0.072
Stride length (cm)	122.36 ± 5.70	133.04 ± 4.37	122.72 ± 7.10	121.08 ± 3.50	23.624, <0.001, 0.383	43.926, <0.001, 0.536	16.907, <0.001, 0.308
Step time (s)	0.65 ± 0.11	0.59 ± 0.15	0.64 ± 0.11	0.60 ± 0.15	4.277, 0.045, 0.101	0.069, 0.794, 0.002	0.007, 0.933, 0.000
Stride time (s)	1.29 ± 0.08	1.18 ± 0.14	1.29 ± 0.08	1.20 ± 0.12	18.405, <0.001, 0.326	0.296, 0.589, 0.008	0.049, 0.825, 0.001
Cadence (steps/min)	97.33 ± 16.28	107.86 ± 28.26	97.84 ± 16.32	105.89 ± 26.13	5.430, 0.025, 0.125	0.096, 0.758, 0.003	0.016, 0.901, 0.000
Total double support (%)	30.48 ± 2.45	27.80 ± 3.97	30.23 ± 5.73	29.70 ± 6.10	1.932, 0.173, 0.048	0.865, 0.358, 0.022	0.706, 0.406, 0.018
Speed (m/s)	1.01 ± 0.28	1.18 ± 0.27	1.00 ± 0.22	1.06 ± 0.27	5.685, 0.022, 0.130	1.242, 0.272, 0.032	0.933, 0.340, 0.024

^1^ dual-task gait training with visual adaptation group; ^2^ control group. Values are expressed as mean ± standard deviation.

**Table 6:** Between-group comparison of adaptive gait parameters in the predictable obstacle walking condition.

	DGTVAG ^1^ (*n* = 20)		CG ^2^ (*n* = 20)		Time (F, *p*, ${\eta p}^{2}$)	Time × Group (F, *p*, ${\eta p}^{2}$)	Group (F, *p*, ${\eta p}^{2}$)
	Pre	Post	Pre	Post			
Step time variability (CV)							
Approach Phase	5.78 ± 1.40	4.22 ± 0.98	5.48 ± 1.28	5.85 ± 1.30	9.845, 0.003, 0.206	23.054, <0.001, 0.378	3.837, 0.058, 0.092
Crossing Phase	4.95 ± 3.50	3.01 ± 2.01	4.40 ± 3.63	5.32 ± 4.75	1.053, 0.311, 0.027	3.549, 0.067, 0.085	0.811, 0.373, 0.021
Post-crossing Phase	6.89 ± 1.40	5.28 ± 0.65	7.19 ± 1.28	5.68 ± 2.52	19.900, <0.001, 0.344	0.021, 0.885, 0.001	0.665, 0.420, 0.017
Step length variability (CV)							
Approach Phase	5.21 ± 1.62	3.51 ± 0.63	6.17 ± 1.43	5.56 ± 1.53	14.601, <0.001, 0.278	8.588, 0.006, 0.184	16.989, <0.001, 0.309
Crossing Phase	7.20 ± 3.86	5.24 ± 1.61	6.18 ± 4.28	6.45 ± 5.02	1.291, 0.263, 0.033	2.588, 0.116, 0.064	0.008, 0.930, 0.000
Post-crossing Phase	7.78 ± 1.62	5.83 ± 0.71	7.28 ± 1.43	6.97 ± 2.82	5.854, 0.020, 0.134	2.406, 0.129, 0.060	0.452, 0.506, 0.012
Step length symmetry (SI)							
Approach Phase	8.37 ± 4.59	7.98 ± 3.31	8.30 ± 5.64	5.42 ± 3.93	4.533, 0.040, 0.107	1.377, 0.248, 0.035	1.184, 0.283, 0.030
Crossing Phase	14.31 ± 10.40	9.49 ± 1.91	11.18 ± 9.59	8.81 ± 7.73	4.955, 0.032, 0.115	0.678, 0.415, 0.018	0.815, 0.372, 0.021
Post-crossing Phase	12.84 ± 4.59	8.41 ± 1.43	12.89 ± 5.64	11.81 ± 6.74	4.897, 0.033, 0.114	1.425, 0.240, 0.036	1.643, 0.208, 0.041
Total double support%							
Approach Phase	31.37 ± 4.23	30.25 ± 4.19	31.67 ± 3.86	32.32 ± 2.03	0.627, 0.433, 0.016	1.998, 0.166, 0.050	1.403, 0.244, 0.036
Crossing Phase	29.56 ± 3.59	26.21 ± 5.44	29.96 ± 5.15	32.62 ± 4.74	2.597, 0.115, 0.064	18.028, <0.001, 0.322	6.853, 0.013, 0.153
Post-crossing Phase	32.01 ± 4.23	29.90 ± 8.47	33.15 ± 3.86	33.06 ± 2.41	0.844, 0.364, 0.022	1.498, 0.229, 0.038	2.384, 0.131, 0.059

^1^ dual-task gait training with visual adaptation group; ^2^ control group. Values are expressed as mean ± standard deviation.

**Table 7:** Between-group comparison of adaptive gait parameters in the unpredictable obstacle walking condition (light).

	DGTVAG ^1^ (*n* = 20)		CG ^2^ (*n* = 20)		Time (F, *p*, ${\eta p}^{2}$)	Time × Group (F, *p*, ${\eta p}^{2}$)	Group (F, *p*, ${\eta p}^{2}$)
	Pre	Post	Pre	Post			
Step time variability (CV)							
Approach Phase	5.84 ± 1.22	5.96 ± 0.88	6.02 ± 1.48	6.55 ± 1.61	3.482, 0.070, 0.084	1.227, 0.275, 0.031	0.908, 0.346, 0.023
Crossing Phase	5.33 ± 3.71	4.31 ± 2.63	5.87 ± 3.68	6.71 ± 3.01	0.020, 0.889, 0.001	3.530, 0.068, 0.085	1.203, 0.280, 0.031
Post-crossing Phase	7.45 ± 1.22	5.09 ± 1.12	7.16 ± 1.48	6.57 ± 2.99	6.800, 0.013, 0.152	2.238, 0.143, 0.056	1.202, 0.280, 0.031
Step length variability (CV)							
Approach Phase	6.82 ± 1.48	5.70 ± 0.95	6.93 ± 1.21	6.32 ± 1.24	9.547, 0.004, 0.201	1.377, 0.248, 0.035	0.712, 0.404, 0.018
Crossing Phase	6.00 ± 3.15	4.02 ± 2.46	7.66 ± 3.88	5.73 ± 1.99	2.074, 0.158, 0.052	0.002, 0.964, 0.000	2.197, 0.146, 0.055
Post-crossing Phase	6.58 ± 1.48	5.90 ± 0.90	6.90 ± 1.21	6.30 ± 3.31	2.119, 0.153, 0.053	0.004, 0.950, 0.000	0.480, 0.493, 0.012
Step length symmetry (SI)							
Approach Phase	8.80 ± 4.52	6.04 ± 4.24	8.21 ± 2.44	7.43 ± 4.10	4.991, 0.031, 0.116	1.220, 0.276, 0.031	0.002, 0.965, 0.000
Crossing Phase	14.76 ± 9.89	10.99 ± 2.30	14.65 ± 10.25	12.57 ± 9.67	1.193, 0.282, 0.030	0.359, 0.553, 0.009	0.269, 0.607, 0.007
Post-crossing Phase	15.76 ± 4.52	11.91 ± 2.29	13.63 ± 2.44	11.61 ± 6.78	5.061, 0.030, 0.118	0.469, 0.498, 0.012	1.466, 0.233, 0.037
Total double support%							
Approach Phase	32.10 ± 4.90	28.92 ± 3.30	29.96 ± 4.57	32.33 ± 3.86	0.114, 0.737, 0.003	6.060, 0.018, 0.137	0.768, 0.386, 0.020
Crossing Phase	30.67 ± 7.50	29.40 ± 5.99	32.52 ± 7.88	32.27 ± 5.07	0.702, 0.407, 0.018	0.200, 0.657, 0.005	1.379, 0.247, 0.035
Post-crossing Phase	31.91 ± 4.90	30.88 ± 6.26	32.41 ± 4.57	31.18 ± 1.77	0.066, 0.799, 0.002	0.013, 0.910, 0.000	0.070, 0.792, 0.002

^1^ dual-task gait training with visual adaptation group; ^2^ control group. Values are expressed as mean ± standard deviation.

**Table 8:** Between-group comparison of adaptive gait parameters in the unpredictable obstacle walking condition (noise).

	DGTVAG ^1^ (*n* = 20)		CG ^2^ (*n* = 20)		Time (F, *p*, ${\eta p}^{2}$)	Time × Group (F, *p*, ${\eta p}^{2}$)	Group (F, *p*, ${\eta p}^{2}$)
	Pre	Post	Pre	Post			
Step time variability (CV)							
Approach Phase	5.44 ± 1.35	4.68 ± 1.17	5.64 ± 1.31	6.05 ± 1.54	0.665, 0.420, 0.017	7.435, 0.010, 0.164	4.533, 0.040, 0.107
Crossing Phase	5.51 ± 3.56	3.93 ± 2.01	6.15 ± 4.10	6.55 ± 4.14	1.048, 0.313, 0.027	2.930, 0.095, 0.072	2.826, 0.101, 0.069
Post-crossing Phase	8.26 ± 1.35	4.28 ± 0.64	7.79 ± 1.31	6.93 ± 3.33	47.800, <0.001, 0.557	19.957, <0.001, 0.344	4.653, 0.037, 0.109
Step length variability (CV)							
Approach Phase	5.52 ± 1.54	5.56 ± 1.37	5.74 ± 1.35	5.38 ± 1.02	0.536, 0.469, 0.014	0.883, 0.353, 0.023	0.003, 0.957, 0.000
Crossing Phase	6.93 ± 3.89	4.16 ± 2.16	6.27 ± 5.18	5.42 ± 3.47	8.083, 0.007, 0.175	2.290, 0.139, 0.057	0.084, 0.774, 0.002
Post-crossing Phase	7.97 ± 1.54	4.76 ± 0.83	7.77 ± 1.35	8.01 ± 3.19	18.744, <0.001, 0.330	25.059, <0.001, 0.397	8.966, 0.005, 0.191
Step length symmetry (SI)							
Approach Phase	9.48 ± 3.64	5.86 ± 3.13	8.94 ± 2.96	5.35 ± 3.27	48.519, <0.001, 0.561	0.001, 0.979, 0.000	0.351, 0.557, 0.009
Crossing Phase	15.61 ± 10.84	8.38 ± 3.52	12.22 ± 10.89	15.19 ± 9.92	1.811, 0.186, 0.045	10.372, 0.003, 0.214	0.473, 0.496, 0.012
Post-crossing Phase	13.04 ± 3.64	9.83 ± 3.06	11.42 ± 2.96	12.50 ± 7.78	1.584, 0.216, 0.040	6.413, 0.016, 0.144	0.173, 0.680, 0.005
Total double support%							
Approach Phase	32.42 ± 4.78	29.60 ± 4.22	32.38 ± 3.18	31.53 ± 4.17	7.749, 0.008, 0.169	2.218, 0.145, 0.055	0.696, 0.409, 0.018
Crossing Phase	31.51 ± 5.94	27.63 ± 5.21	29.63 ± 7.43	30.98 ± 7.14	1.515, 0.226, 0.038	6.467, 0.015, 0.145	0.170, 0.682, 0.004
Post-crossing Phase	29.76 ± 4.78	28.49 ± 2.25	31.16 ± 3.18	32.00 ± 7.55	0.063, 0.803, 0.002	1.483, 0.231, 0.038	3.728, 0.061, 0.089

^1^ dual-task gait training with visual adaptation group; ^2^ control group. Values are expressed as mean ± standard deviation.

**Table 9:** Between-group comparison of balance.

	DGTVAG ^1^ (*n* = 20)		CG ^2^ (*n* = 20)		Time (F, *p*, ${\eta p}^{2}$)	Time × Group (F, *p*, ${\eta p}^{2}$)	Group (F, *p*, ${\eta p}^{2}$)
	Pre	Post	Pre	Post			
BBS ^3^	45.55 ± 2.33	50.00 ± 2.15	46.05 ± 2.21	46.70 ± 2.99	313.771, <0.001, 0.892	174.197, <0.001, 0.821	3.398, 0.073, 0.082

^1^ dual-task gait training with visual adaptation group; ^2^ control group; ^3^ Berg balance scale. Values are expressed as mean ± standard deviation.

**Table 10:** Between-group comparison of executive function.

	DGTVAG ^1^ (*n* = 20)		CG ^2^ (*n* = 20)		Time (F, *p*, ${\eta p}^{2}$)	Time × Group (F, *p*, ${\eta p}^{2}$)	Group (F, *p*, ${\eta p}^{2}$)
	Pre	Post	Pre	Post			
Stroop test							
Accuracy (%)	79.17 ± 3.61	84.42 ± 3.35	78.33 ± 3.63	79.00 ± 3.35	224.234, <0.001, 0.855	134.576, <0.001, 0.780	8.307, 0.006, 0.179
Time (s)	69.40 ± 6.73	58.95 ± 4.54	66.35 ± 4.09	66.49 ± 4.21	275.752, <0.001, 0.879	290.376, <0.001, 0.884	2.090, 0.156, 0.052
Trail Making Test							
Part A (s)	47.81 ± 5.32	41.30 ± 3.91	46.06 ± 3.37	46.86 ± 3.59	173.281, <0.001, 0.820	285.095, <0.001, 0.882	2.206, 0.146, 0.055
Part B (s)	98.87 ± 7.04	87.82 ± 5.85	99.82 ± 3.69	100.21 ± 4.04	509.802, <0.001, 0.931	586.708, <0.001, 0.939	15.971, <0.001, 0.296

^1^ dual-task gait training with visual adaptation group; ^2^ control group. Values are expressed as mean ± standard deviation.

**Table 11:** Between-group comparison of fall efficacy.

	DGTVAG ^1^ (*n* = 20)		CG ^2^ (*n* = 20)		Time (F, *p*, ${\eta p}^{2}$)	Time × Group (F, *p*, ${\eta p}^{2}$)	Group (F, *p*, ${\eta p}^{2}$)
	Pre	Post	Pre	Post			
FES-I ^3^	28.55 ± 2.33	23.75 ± 2.31	26.95 ± 3.47	26.65 ± 3.72	509.474, <0.001, 0.931	396.649, <0.001, 0.913	0.468, 0.498, 0.012

^1^ dual-task gait training with visual adaptation group; ^2^ control group; ^3^ Falls Efficacy Scale-International. Values are expressed as mean ± standard deviation.

## Data Availability

The data presented in this study are not publicly available due to privacy and ethical restrictions, as they contain potentially identifiable participant information. The data are available from the corresponding author upon reasonable request and subject to institutional approval.

## Abbreviations

The following abbreviations are used in this manuscript:

MCI	Mild cognitive impairment
DGTVA	Dual-task gait training with visual adaptation
DGTVAG	Dual-task gait training with visual adaptation group
CG	Control group
MoCA-K	Montreal Cognitive Assessment-Korean version
BMI	Body mass index
BBS	Berg Balance Scale
K-CWST	Korean Color-Word Stroop Test
K-TMT-e	Korean Trail Making Test for the Elderly
FES-I	Falls Efficacy Scale-International
CV	Coefficient of variation
SI	Symmetry index
IRB	Institutional Review Board
CRIS	Clinical Research Information Service
